# To Which Mr. H. E. Smithers Replies as Follows

**Published:** 1920-02-21

**Authors:** H. E. Smithers

**Affiliations:** General Manager.


**To which Mr. H. E. Smithers replies as follows:**

Sir,—Mr. Danvers Power's letter in *The Times* of February 17 is calculated to cloud the issue. Sir Henry Burdett originated the Uniform System, and whatever the elaboration necessary to the growth of hospitals, the fact remains.

The glory of James Watt does not fade before the steam engine of to-day,

"A chaque saint sa chandelle."

Yours faithfully,
For the Scientific Press, Ltd.,
H. E. Smithers,
General Manager.

